# Association of the hs-CRP-TyG index with coronary artery disease risk and angiographic severity: a retrospective comparative study with the TyG index

**DOI:** 10.3389/fendo.2026.1836149

**Published:** 2026-05-07

**Authors:** Min Yang, Ling-yun Hu, Tao Liu, Yuan-yuan Yi, Yun-hu Xu

**Affiliations:** 1 Department of General Practice, Chongqing University Fuling Hospital, Chongqing University, Chongqing, China; 2 Department of Neurology, Fuling People's Hospital of Chongqing, Chongqing, China

**Keywords:** coronary artery disease, high-sensitivity C-reactive protein, hs-CRP-TyG index, inflammation, risk prediction, triglyceride–glucose index

## Abstract

**Objective:**

Inflammation and insulin resistance synergistically contribute to the progression of coronary artery disease (CAD), yet the predictive performance of single biomarkers remains limited. This study aimed to evaluate the predictive value of a composite index derived from high-sensitivity C-reactive protein (hs-CRP) and the triglyceride–glucose (TyG) index, termed the hs-CRP-TyG index, and to assess its potential incremental value compared with the TyG index alone.

**Methods:**

A total of 1,372 patients who underwent coronary angiography were retrospectively enrolled, including 1,190 patients with CAD and 182 without CAD. The hs-CRP-TyG index was calculated for each participant. Multivariable logistic regression analysis was performed to evaluate associations with CAD risk and severity. Model performance was assessed using receiver operating characteristic (ROC) curves with DeLong test, calibration analysis, and bootstrap internal validation (1,000 resamples) to estimate optimism-corrected performance. Restricted cubic spline (RCS) analysis was used to explore nonlinear dose–response relationships. Decision curve analysis (DCA) was conducted to evaluate clinical utility. Incremental predictive value over the TyG index was assessed using net reclassification improvement (NRI) and integrated discrimination improvement (IDI).

**Results:**

The hs-CRP-TyG level was significantly higher in the CAD group than in the non-CAD group [1.45 (1.02–1.90) vs. 1.23 (0.89–1.69), *P* < 0.01]. In the fully adjusted model (Model 4), the hs-CRP-TyG index was independently associated with CAD risk (OR = 1.34, 95% *CI*: 1.03–1.78, *P* < 0.05) and showed a graded positive association with severe coronary lesions. RCS analysis revealed a significant nonlinear dose–response relationship, with an accelerated increase in CAD risk when hs-CRP-TyG exceeded 1.42. In direct comparison, the hs-CRP-TyG index demonstrated higher sensitivity than the TyG index alone (68% vs. 47%), whereas overall discriminative performance was comparable (AUC: 0.79 vs. 0.79; DeLong test *P* > 0.05). Reclassification analyses (NRI and IDI) suggested limited incremental predictive value after full adjustment. DCA indicated a potential net clinical benefit within certain threshold probabilities. Subgroup analyses confirmed the robustness of these associations, with a stronger effect observed among patients with impaired cardiac function.

**Conclusion:**

The hs-CRP-TyG index is associated with both the presence and severity of CAD and reflects the combined effects of metabolic and inflammatory processes. Although it demonstrates higher sensitivity than the TyG index alone, its overall discriminative performance is comparable, and its incremental predictive value appears limited after full adjustment. This composite marker may have limited complementary value in high-risk settings, but further prospective validation is warranted.

## 1 Introduction

Coronary artery disease (CAD) remains the leading cause of mortality and disability worldwide (1), with coronary atherosclerosis constituting its fundamental pathological substrate (2). Although lipid-lowering therapy centered on low-density lipoprotein cholesterol (LDL-C) has substantially improved clinical outcomes, a significant burden of residual cardiovascular risk persists in routine practice. A considerable proportion of patients continue to experience major adverse cardiovascular events or disease progression despite achieving recommended LDL-C targets (3). This observation suggests that mechanisms beyond lipid dysregulation play critical roles in the initiation and progression of atherosclerosis (4), underscoring the need to identify novel biomarkers to enable more precise risk stratification.

Chronic low-grade inflammation and insulin resistance (IR) are closely interconnected and mutually reinforcing processes that jointly accelerate atherosclerotic plaque formation, progression, and destabilization (5–7). Systemic inflammatory indices have been shown to consistently predict atherosclerotic risk across diverse populations, including community-based cohorts and critically ill patients, and their elevation has been closely associated with plaque development, providing quantifiable clinical evidence for the “inflammation–IR–plaque” axis (8). Within this pathological context, high-sensitivity C-reactive protein (hs-CRP), a sensitive marker of chronic low-grade systemic inflammation, has been confirmed in a large-scale meta-analysis including 160,309 individuals without prior vascular disease to be significantly associated with incident CAD and to serve as an independent predictor of risk (9). Compared with conventional CRP, hs-CRP enables the precise detection of low-grade inflammatory states and more accurately reflects the long-term pathological process of atherosclerosis. The triglyceride–glucose (TyG) index, a convenient and reliable surrogate marker of IR, has demonstrated strong concordance with the hyperinsulinemic–euglycemic clamp, the reference standard for IR assessment. It has been consistently associated with the extent and severity of coronary lesions as well as adverse cardiovascular outcomes, thereby providing a practical tool for CAD risk stratification (10). However, most existing studies have evaluated inflammatory or metabolic markers in isolation, largely overlooking their pathophysiological synergy. Theoretically, a composite index integrating chronic inflammation (hs-CRP) and metabolic dysfunction (TyG)—the hs-CRP-TyG index—may better reflect the interplay between inflammatory and metabolic pathways in atherosclerosis, thereby providing a more comprehensive assessment of CAD risk compared with either marker alone.

Although the hs-CRP-TyG index has demonstrated independent prognostic value in ischemic stroke (11), depression risk prediction (12), and chronic kidney disease, including peritoneal dialysis populations (13), its application in CAD remains insufficiently characterized. Current cardiovascular studies have primarily focused on its binary association with CAD occurrence (14) or have reported preliminary correlations with three-vessel disease (TVD) in small samples (15). Large-scale investigations based on coronary angiography (CAG), the diagnostic gold standard, are lacking, particularly those systematically quantifying the nonlinear dose–response relationship between hs-CRP-TyG and angiographic severity of CAD, including stenosis grading and left main involvement.

In addition, previous studies have shown limitations in confounder adjustment, often neglecting the potential influence of peripheral arterial disease and cardiac systolic function on the metabolic–inflammatory pathway, thereby introducing possible residual confounding bias. More importantly, whether the hs-CRP-TyG index provides incremental predictive value over the TyG index alone—especially in terms of model efficiency and sensitivity across different levels of model complexity—remains uncertain. Addressing these gaps is therefore of clinical relevance.

Accordingly, the present study was designed to fill these knowledge gaps using a large retrospective cohort. The objectives were: (1) to determine the independent association and nonlinear dose–response relationship between the hs-CRP-TyG index and both CAD occurrence and severe stenosis grading; (2) to evaluate the robustness of these associations after comprehensive adjustment for multidimensional covariates, including ankle–brachial index (ABI), cardiac function, and traditional cardiovascular risk factors; and (3) to explore the potential incremental value of the hs-CRP-TyG index over the TyG index alone in terms of sensitivity.

This study aims to provide a practical tool for risk assessment in high-risk clinical settings and individualized risk stratification in CAD, with particular emphasis on identifying clinically significant disease rather than minimal early lesions.

## 2 Materials and methods

### 2.1 Study design and population

This study was conducted as a single-center retrospective cohort using the hospital electronic medical record system. Patients who were admitted for chest pain or related symptoms and underwent coronary angiography (CAG) between January 2022 and December 2024 were consecutively enrolled. The study protocol was approved by the Ethics Committee of Fuling Hospital, Chongqing University (Approval No.: 2026CDFSFLYYEC-021), and written informed consent was waived due to the retrospective design. All data were de-identified to ensure patient privacy (16).

Inclusion criteria were as follows:

1. Age ≥ 18 years;
2. Admission for suspected CAD and completion of CAG;
3. Complete clinical data, with key variables missing in < 10% of cases.

Exclusion criteria were:

1. Patients in the acute phase of acute myocardial infarction (AMI) (17);
2. Presence of acute infection, trauma, or other acute stress conditions at admission;
3. Severe hepatic or renal dysfunction (eGFR < 30 mL/min/1.73 m²), malignancy, or autoimmune disease;
4. Missing key biochemical parameters (e.g., hs-CRP, glucose, lipid profile) that precluded the calculation of core indices.
5. History of coronary artery bypass grafting (CABG).

### 2.2 Data collection and definitions

Baseline characteristics, laboratory results, and imaging data were retrospectively extracted from the electronic medical record system.

#### 2.2.1 Core predictor: hs-CRP-TyG index

The primary exposure variable was the hs-CRP-TyG index, calculated as: hs-CRP-TyG =ln [hs-CRP (mg/L) ×TyG]. hs-CRP and TyG were log-transformed due to right-skewed distributions to improve model stability.

where the TyG index was defined as: TyG = ln [TG (mg/dL) × FBG (mg/dL)/2] (18).

All laboratory measurements were obtained from the first fasting venous blood sample collected within 24 hours of admission. Biochemical assays were performed using standard enzymatic methods, and hs-CRP was measured by high-sensitivity immunoturbidimetry in an ISO-certified central laboratory under strict quality control procedures.

Based on the cohort distribution, hs-CRP-TyG quartiles were defined as follows: T1 < 1.000, T2 ≥ 1.000 and < 1.420, T3 ≥ 1.420 and < 1.880, and T4 ≥ 1.880. The traditional TyG index was also calculated for comparison.

#### 2.2.2 Covariates

All covariates were extracted within the same baseline window (admission ±24 h):

Demographics and lifestyle: age, sex, body mass index (BMI), smoking, and alcohol consumption.

Medical history and medications: hypertension, diabetes mellitus, prior CAD, atrial fibrillation, dyslipidemia, stroke history, and pre-admission statin use.

Laboratory indices: full lipid panel, uric acid, hs-CRP, hematology-derived indices (e.g., lymphocyte ratio), and fibrinogen.

Cardiac function and vascular assessment:

Left ventricular ejection fraction (EF): measured by experienced echocardiographers using the biplane Simpson method, averaging three cardiac cycles, according to ASE guidelines (19).

Ankle–brachial index (ABI): measured using a Doppler device according to standard protocols (20).

#### 2.2.3 Outcome definitions

Primary outcomes were independently adjudicated by two cardiologists at the associate chief physician level or above, with discrepancies resolved by a third expert (21):

1. CAD occurrence: defined as ≥50% stenosis in at least one major coronary artery or its primary branch on CAG.
2. CAD severity: graded by the number of diseased vessels:
L0: no stenosis or < 50%;
L1: single-vessel disease;
L2: two-vessel disease;
L3: three-vessel disease or left main involvement.

All CAG results were assessed using standard projections, supplemented by quantitative coronary angiography (QCA) when necessary to ensure accurate stenosis evaluation. Outcome assessment was strictly performed after baseline measurements.

### 2.3 Statistical analysis

All analyses were performed using R version 4.3.1. A two-sided *P* < 0.05 was considered statistically significant. Continuous variables were expressed as mean ± standard deviation (SD) or median (interquartile range, IQR) and compared using the independent t-test or Mann–Whitney U test, as appropriate. Categorical variables were presented as frequencies and percentages and compared using the chi-square test or Fisher’s exact test.

Variable selection was based on a combination of clinical relevance, univariable logistic regression (P < 0.10), and random forest importance ranking to improve robustness. Multicollinearity was assessed using the variance inflation factor (VIF), where a VIF > 5 was considered indicative of significant multicollinearity and excluded. Selected variables were entered into multivariable logistic regression models. To mitigate potential instability introduced by data-driven variable selection, bootstrap resampling was additionally used to assess model robustness.

Four sequential models were constructed to evaluate the association between the hs-CRP-TyG index and CAD: Model 1 (unadjusted); Model 2 (adjusted for age, sex, smoking, and alcohol consumption); Model 3 (further adjusted for hypertension, diabetes mellitus, and lipid-lowering therapy); and Model 4 (additionally adjusted for uric acid, fasting glucose, lymphocyte ratio, fibrinogen, and HDL-C).

Restricted cubic spline (RCS) analysis was employed to investigate potential nonlinear relationships. Model performance was evaluated using the area under the receiver operating characteristic curve (AUC), with comparisons performed using DeLong’s test. Calibration was assessed using calibration plots and the Hosmer–Lemeshow goodness-of-fit test. To assess internal validity and model stability, bootstrap resampling (with 1,000 iterations) was performed to estimate the optimism-corrected performance (C-index). The incremental predictive value of the hs-CRP-TyG index over the TyG index was assessed using net reclassification improvement (NRI) and integrated discrimination improvement (IDI) based on nested models.

Subgroup analyses were performed across prespecified strata, and interactions were tested using multiplicative terms.

## 3 Results

### 3.1 Baseline characteristics

A total of 1,372 patients were included, of whom 1,190 (86.7%) had CAD and 182 (13.3%) did not (**Table 1**). Compared with the non-CAD group, the CAD group had a higher proportion of males (51.0% vs. 35.2%, *P* < 0.01). No significant differences were observed between groups in overall smoking or alcohol consumption patterns (*P* > 0.05), although the proportion of frequent alcohol drinkers was numerically higher in the CAD group (9.9% vs. 5.0%). Regarding medical history, patients in the CAD group had significantly higher prevalence of hypertension (51.8% vs. 25.3%), diabetes mellitus (19.8% vs. 4.4%), prior CAD (22.3% vs. 6.6%), dyslipidemia (16.6% vs. 7.7%), stroke history (8.9% vs. 1.7%), and pre-admission lipid-lowering therapy (32.2% vs. 12.2%) compared with the non-CAD group (all *P* < 0.01). History of prior stent implantation was also more common in the CAD group (8.4% vs. 1.1%, *P* < 0.01). Biochemical profiles demonstrated marked inflammatory activation and metabolic dysregulation in CAD patients. The hs-CRP-TyG index was significantly elevated [1.45 (1.02–1.90) vs. 1.23 (0.89–1.69), *P* < 0.01]. Inflammatory and hematological markers, including white blood cell count, neutrophil ratio, fibrinogen, D-dimer, and hs-CRP, were significantly higher, whereas platelet count [207 (170–251) vs. 220 (182–261) ×10^9^/L, *P* < 0.05] and lymphocyte ratio were significantly lower (all *P* < 0.01). Metabolic and other laboratory indices showed higher fasting glucose, uric acid, creatinine, and NT-proBNP levels in the CAD group, while HDL-C was significantly lower (all *P* < 0.05). Total cholesterol (TC) and LDL-C were slightly lower in CAD patients (*P* < 0.05). Additionally, FT3 and TSH levels were lower, whereas FT4 was higher in the CAD group (all *P* < 0.05).

**Table 1:** Baseline clinical characteristics of study participants according to coronary artery disease status.

Variable	ALL (n=1,372)	No CAD (n=182)	CAD (n=1,190)	*H / X^2^* value	*P* value
hs-CRP-TyG	1.42 (1.00 - 1.88)	1.23 (0.89 - 1.69)	1.45 (1.02 - 1.90)	14.05	<0.01
Gender					
Male	671 (48.9%)	64 (35.2%)	607 (51.0%)	15.86	<0.01
Female	701 (51.1%)	118 (64.8%)	583 (49.0%)		
Smoking history					
Never	1008 (73.5%)	140 (76.9%)	868 (72.9%)	1.32	>0.05
Occasional	38 (2.77%)	4 (2.20%)	34 (2.86%)		
Current	326 (23.8%)	38 (20.9%)	288 (24.2%)		
Drinking history					
Never	1070 (78.0%)	151 (83.0%)	919 (77.2%)	4.95	>0.05
Occasional	175 (12.8%)	22 (12.1%)	153 (12.9%)		
Current	127 (9.26%)	9 (4.95%)	118 (9.92%)		
Height (cm)	160 (154 - 165)	158 (155 - 165)	160 (153 - 165)	0.35	>0.05
Weight (kg)	62.0 (55.0 - 70.0)	63.0 (56.1 - 70.0)	62.0 (55.0 - 70.0)	0.34	>0.05
BMI	24.6 (22.2 - 26.9)	24.8 (22.6 - 27.1)	24.5 (22.2 - 26.9)	1.17	>0.05
WBC	6.34 (5.20 - 7.87)	6.04 (4.88 - 7.20)	6.39 (5.25 - 7.94)	8.28	<0.01
Hb	136 (126 - 147)	135 (127 - 147)	137 (126 - 147)	0.17	>0.05
PLT	208 (172 - 252)	220 (182 - 261)	207 (170 - 251)	5.01	<0.05
Neutrophil ratio	64.6 (57.6 - 72.0)	62.5 (54.7 - 69.5)	64.9 (57.9 - 72.3)	10.36	<0.01
Lymphocyte ratio	25.5 (18.6 - 31.8)	28.0 (21.3 - 34.7)	25.2 (18.2 - 31.3)	12.97	<0.01
Fibrinogen	2.96 (2.53 - 3.56)	2.74 (2.38 - 3.22)	2.99 (2.57 - 3.64)	23.46	<0.01
D-dimer	0.32 (0.19 - 0.63)	0.26 (0.19 - 0.42)	0.34 (0.19 - 0.68)	13.54	<0.01
Cardiac troponin	0.01 (0.01 - 0.01)	0.01 (0.01 - 0.01)	0.01 (0.01 - 0.01)	0.09	>0.05
NT-proBNP	128 (71.0 - 318)	102 (58.0 - 248)	132 (74.0 - 324)	8.65	<0.01
FT3	4.68 (4.16 - 5.24)	4.84 (4.38 - 5.41)	4.66 (4.12 - 5.23)	7.99	<0.01
FT4	13.2 (11.1 - 15.6)	12.6 (10.8 - 15.1)	13.2 (11.2 - 15.6)	6.38	<0.05
TSH	1.68 (1.14 - 2.62)	2.05 (1.31 - 3.02)	1.65 (1.12 - 2.55)	10.24	<0.01
TC	4.61 (3.89 - 5.34)	4.78 (4.23 - 5.35)	4.56 (3.83 - 5.34)	4.99	<0.05
TG	1.31 (0.95 - 1.97)	1.30 (0.95 - 1.85)	1.31 (0.95 - 1.98)	0.92	>0.05
LDL	2.91 (2.18 - 3.53)	3.05 (2.50 - 3.65)	2.89 (2.16 - 3.51)	5.74	<0.05
HDL	1.16 (0.98 - 1.40)	1.21 (1.05 - 1.47)	1.15 (0.98 - 1.39)	9.23	<0.01
Lp(a)	89.9 (49.7 - 217)	82.7 (50.2 - 169)	92.0 (49.7 - 226)	1.76	>0.05
hs-CRP	1.75 (0.80 - 6.30)	1.31 (0.60 - 3.60)	1.80 (0.80 - 6.80)	11.08	<0.01
Cr	67.0 (56.0 - 80.4)	62.8 (55.1 - 73.0)	67.9 (56.2 - 81.2)	13.7	<0.01
UA	321 (266 - 386)	303 (247 - 356)	324 (269 - 394)	10.88	<0.01
FBG	5.39 (4.80 - 6.33)	5.08 (4.69 - 5.65)	5.47 (4.84 - 6.50)	23.85	<0.01
ALT	18.4 (13.5 - 26.5)	18.0 (13.6 - 26.9)	18.4 (13.5 - 26.5)	0.01	>0.05
AST	20.8 (17.2 - 26.4)	20.5 (17.4 - 25.6)	21.0 (17.2 - 26.4)	0.37	>0.05
Pre-admission lipid-lowering medication					
No	959 (70.4%)	158 (87.8%)	801 (67.8%)	30.03	<0.01
Yes	403 (29.6%)	22 (12.2%)	381 (32.2%)		
Hypertension					
No	709 (51.7%)	136 (74.7%)	573 (48.2%)	44.64	<0.01
Yes	663 (48.3%)	46 (25.3%)	617 (51.8%)		
DM history					
No	1128 (82.2%)	174 (95.6%)	954 (80.2%)	25.73	<0.01
Yes	244 (17.8%)	8 (4.40%)	236 (19.8%)		
CAD history					
No	1095 (79.8%)	170 (93.4%)	925 (77.7%)	23.92	<0.01
Yes	277 (20.2%)	12 (6.59%)	265 (22.3%)		
AF history					
No	1331 (97.0%)	179 (98.4%)	1152 (96.8%)	1.30	>0.05
Yes	41 (2.99%)	3 (1.65%)	38 (3.19%)		
Hyperlipidemia					
No	1161 (84.6%)	168 (92.3%)	993 (83.4%)	9.53	<0.01
Yes	211 (15.4%)	14 (7.69%)	197 (16.6%)		
Coronary stenting					
No	1270 (92.6%)	180 (98.9%)	1090 (91.6%)	12.24	<0.01
Yes	102 (7.43%)	2 (1.10%)	100 (8.40%)		
History of stroke					
No	1263 (92.1%)	179 (98.4%)	1084 (91.1%)	11.37	<0.01
Yes	109 (7.94%)	3 (1.65%)	106 (8.91%)		
Fatty liver disease					
No	993 (72.4%)	140 (76.9%)	853 (71.7%)	2.12	>0.05
Yes	378 (27.6%)	42 (23.1%)	336 (28.3%)		
Echocardiographic EF.					
Reduced	66 (4.81%)	13 (7.14%)	53 (4.45%)	2.49	>0.05
Normal	1306 (95.2%)	169 (92.9%)	1137 (95.5%)		
ABI vascular function assessment					
Normal	1290 (94.0%)	177 (97.3%)	1113 (93.5%)	3.89	>0.05
Abnormal	82 (5.98%)	5 (2.75%)	77 (6.47%)		

hs-CRP-TyG, hs-CRP–Triglyceride-Glucose index; BMI, Body mass index; WBC, White blood cell; Hb, Hemoglobin; PLT, Platelet; NT-proBNP, N-terminal pro-B-type natriuretic peptide; FT3, Free triiodothyronine; FT4, Free thyroxine; TSH, Thyroid-stimulating hormone; TC, Total cholesterol; TG, Triglyceride; LDL-C, Low-density lipoprotein cholesterol; HDL-C, High-density lipoprotein cholesterol; Lp(a), Lipoprotein(a); hs-CRP, High-sensitivity C-reactive protein; Cr, Creatinine; UA, Uric acid; FBG, Fasting blood glucose; ALT, Alanine aminotransferase; AST, Aspartate aminotransferase; DM, Diabetes mellitus; CAD, Coronary artery disease; AF, Atrial fibrillation; ABI, ankle-brachial index; EF, Ejection fraction. Categorical variables are presented as number and percentage (n, %), and continuous variables are presented as median (interquartile range, IQR).

### 3.2 Variable selection and importance assessment

Univariable logistic regression demonstrated that hs-CRP-TyG was positively associated with CAD risk (OR = 1.63, 95% CI: 1.29–2.09, *P* < 0.001). Traditional risk factors, including age, sex, hypertension, diabetes, and fibrinogen, were also significantly associated (all *P* < 0.05), whereas LDL-C and TC showed no positive association, possibly due to higher statin use in the CAD group.

Random forest modeling further highlighted the nonlinear predictive contribution of variables. Based on consistent rankings by mean decrease in accuracy and node impurity, age ranked first, and hs-CRP-TyG consistently ranked third, confirming its robustness within complex models. Neutrophil ratio, lymphocyte ratio, and HDL-C had higher importance than traditional lipid markers, emphasizing the key role of inflammatory cell distribution and lipid metabolism in CAD prediction. Fasting glucose, TSH, NT-proBNP, and fibrinogen were also identified as high-importance variables.

Combining univariable analysis (*P* < 0.1) and random forest rankings, age, sex, hs-CRP-TyG, relevant clinical history, and the aforementioned high-importance biochemical markers were included in the subsequent multivariable models. Variance inflation factors (VIF) for all included variables were <5, indicating no multicollinearity and suitability for regression analysis.

### 3.3 Association between hs-CRP-TyG and CAD risk and severity

#### 3.3.1 Independent association with CAD occurrence

Multivariable logistic regression indicated that hs-CRP-TyG was an independent risk factor for CAD (**Table 2**). In the fully adjusted model (Model 4), per unit increase (log-transformed scale) in hs-CRP-TyG was associated with a 34% higher risk of CAD (OR = 1.34, 95% *CI*: 1.03–1.78, *P* < 0.05), independent of traditional risk factors.

**Table 2:** Association between hs-CRP-TyG index and the risk and severity of coronary artery disease.

Variable	CAD incidence risk OR (95% *CI*), *P*-value	CAD severity OR (95% *CI*), *P*-value
Model 1		
hs-CRP-TyG (Continuous, per unit)	1.63 (1.29 – 2.09), <0.01	
Quartiles of hs-CRP-TyG		Severity Grades
T1 (Reference)	Reference	L-0 (Reference)
T2	1.18 (0.69 – 2.01), >0.05	L-1: 1.22 (0.90 – 1.68), >0.05
T3	0.91 (0.43 – 1.86), >0.05	L-2: 1.49 (1.17 – 1.91), <0.01
T4	0.77 (0.24 – 2.33), >0.05	L-3: 1.76 (1.34 – 2.33), <0.01
Model 2		
hs-CRP-TyG (Continuous, per unit)	1.75 (1.37 – 2.25), <0.01	
Quartiles of hs-CRP-TyG		Severity Grades
T1 (Reference)	Reference	L-0 (Reference)
T2	0.95 (0.53 – 1.68), >0.05	L-1: 1.35 (0.97 – 1.89), >0.05
T3	0.87 (0.39 – 1.84), >0.05	L-2: 1.59 (1.25 – 2.06), <0.01
T4	0.68 (0.20 – 2.19), >0.05	L-3: 2.06 (1.51 – 2.85), <0.01
Model 3		
hs-CRP-TyG (Continuous, per unit)	1.51 (1.18 – 1.96), <0.01	
Quartiles of hs-CRP-TyG		Severity Grades
T1 (Reference)	Reference	L-0 (Reference)
T2	0.99 (0.55 – 1.74), >0.05	L-1: 1.14 (0.80 – 1.62), >0.05
T3	0.85 (0.39 – 1.81), >0.05	L-2: 1.42 (1.10 – 1.84), <0.01
T4	0.67 (0.20 – 2.14), >0.05	L-3: 1.46 (1.05 – 2.07), <0.05
Model 4		
hs-CRP-TyG (Continuous, per unit)	1.34 (1.03 – 1.78), <0.05	
Quartiles of hs-CRP-TyG		Severity Grades
T1 (Reference)	Reference	L-0 (Reference)
T2	0.98 (0.55 – 1.72), >0.05	L-1: 1.03 (0.70 – 1.52), >0.05
T3	0.83 (0.38 – 1.74), >0.05	L-2: 1.25 (0.95 – 1.67), >0.05
T4	0.68 (0.21 – 2.17), >0.05	L-3: 1.35 (0.94 – 1.96), >0.05

hs-CRP-TyG, hs-CRP–Triglyceride-Glucose index; OR, Odds Ratio; CI, Confidence Interval; T1–T4, Quartiles of hs-CRP-TyG; L-0 to L-3, Severity grades of CAD. Model 1, Unadjusted (Crude model for hs-CRP-TyG). Model 2, Adjusted for Model 1 + Age, Sex, Smoking history, and Alcohol consumption. Model 3, Adjusted for Model 2 + History of Hypertension, History of Diabetes, and Pre-admission lipid-lowering medication use. Model 4, Adjusted for Model 3 + Uric Acid, Lymphocyte-to-Monocyte Ratio, Fibrinogen, HDL-C.

#### 3.3.2 Association with anatomical severity

Multinomial logistic regression demonstrated a graded positive association between hs-CRP-TyG and CAD severity (**Table 2**). With increasing stenosis, OR values rose progressively. After adjusting for major confounders (Model 3), each 1-unit increase in hs-CRP-TyG was associated with a 46% higher risk of extremely severe stenosis (L3) (OR = 1.46, *P* < 0.05), exceeding the risk for severe stenosis (L2, OR = 1.42), and was not significantly associated with mild stenosis (L1). Notably, after additional adjustment for biochemical mediators (Model 4), the association attenuated and lost statistical significance, suggesting partial collinearity or shared pathological pathways with these inflammatory and metabolic markers.

#### 3.3.3 Dose–response relationship

**Figure 1:**
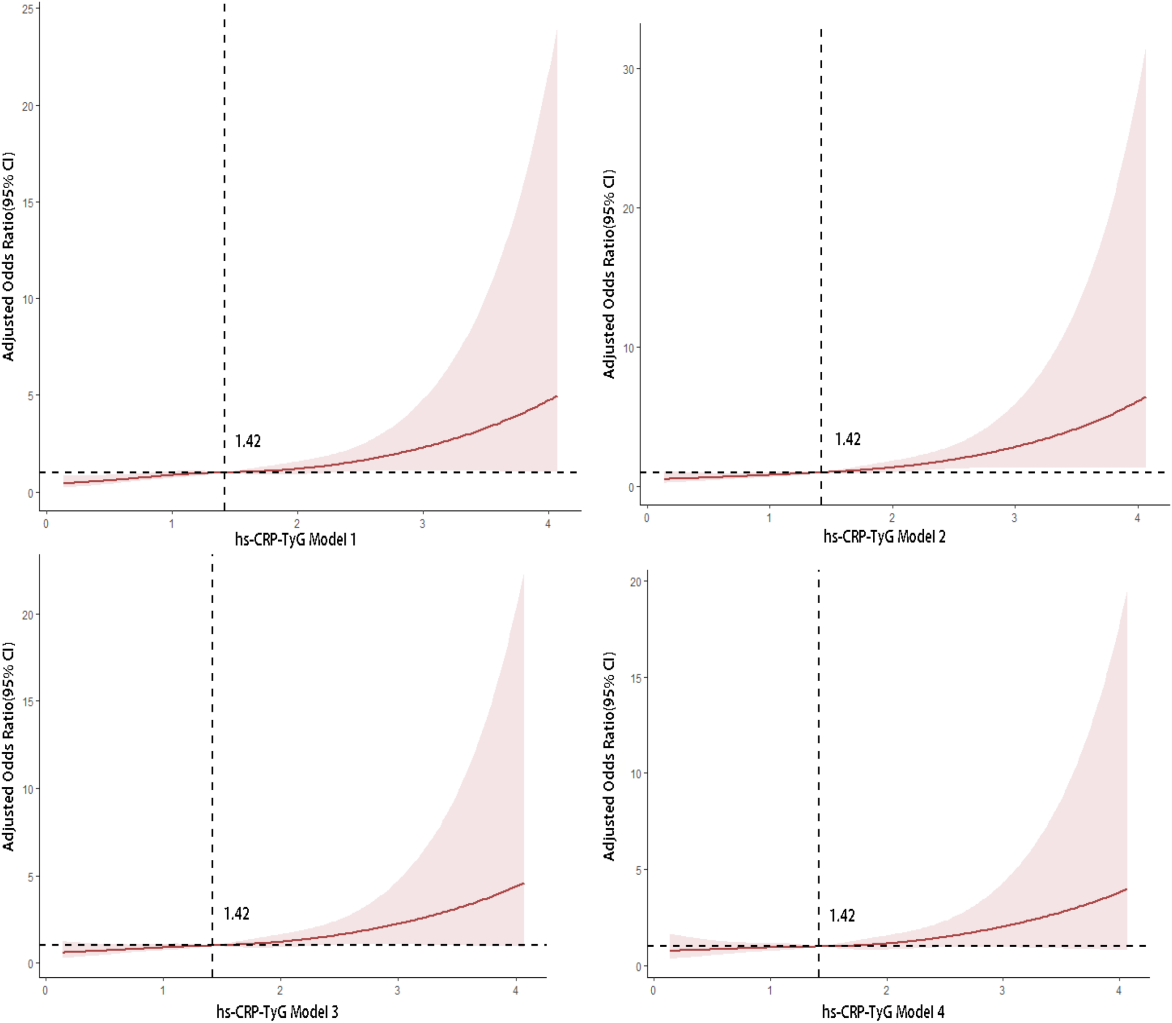
Restricted cubic spline plots showing the nonlinear association between the hs-CRP-TyG index and the risk of coronary artery disease. Solid lines represent the estimated odds ratios (ORs); shaded areas indicate 95% confidence intervals (CIs). The horizontal dashed line indicates OR = 1.0. The clinical interpretation of this threshold effect, where risk significantly increases beyond a value of 1.42, is discussed in detail in the Discussion section. Models 1–4 represent progressively adjusted regression models, ranging from the unadjusted model (Model 1) to the fully adjusted model including all covariates (Model 4).

Restricted cubic spline analysis revealed a significant positive nonlinear association between hs-CRP-TyG and CAD risk (**Figure 1**). Risk increased continuously with hs-CRP-TyG, particularly above 1.42, indicating a potential accelerated risk effect. The inflection point coincided with the lower bound of the third quartile (T3) in this cohort, supporting the observation that moderately elevated levels already confer a sharp increase in risk.

### 3.4 Subgroup analysis and interaction testing

**Figure 2:**
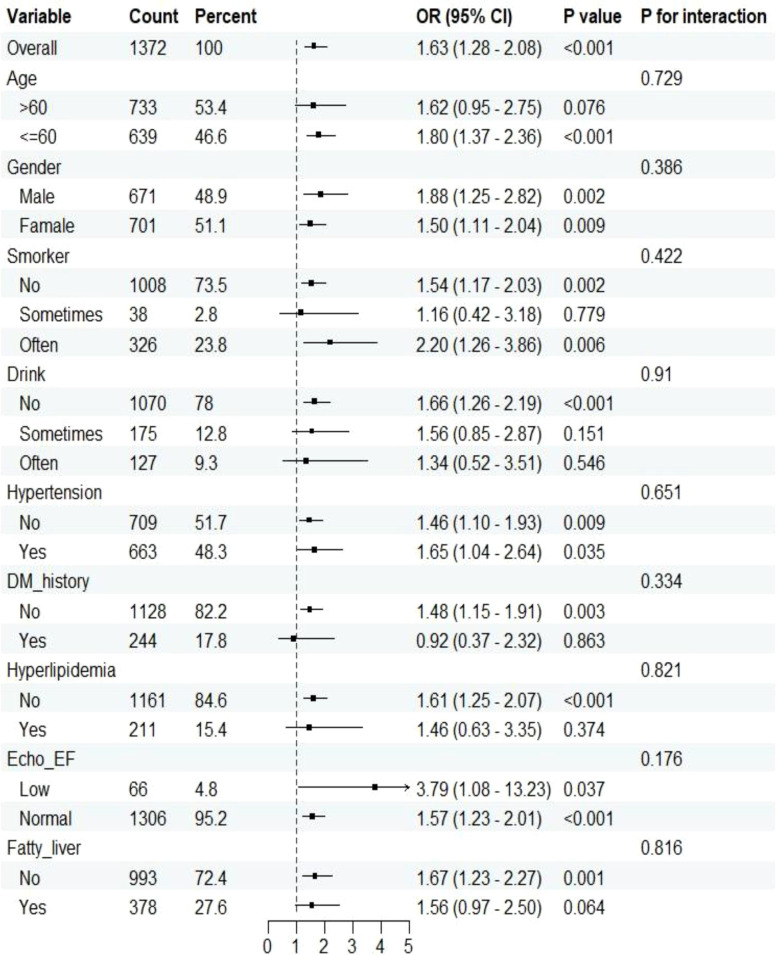
Subgroup analysis of the association between hs-CRP-TyG index and risk of CAD. hs-CRP-TyG, hs-CRP–Triglyceride-Glucose; CAD, coronary artery disease; DM, diabetes mellitus; Echo_EF, echocardiographic ejection fraction; Fatty liver, presence of fatty liver disease; OR, odds ratio; CI, confidence interval. Note: All models were adjusted for age, sex, smoking status, alcohol consumption, hypertension, diabetes, lipid-lowering medication use, uric acid, lymphocyte ratio, fibrinogen, and HDL-C unless otherwise specified in subgroup stratification.

Forest plots (**Figure 2**) showed that the positive association between hs-CRP-TyG and CAD risk remained consistent across predefined subgroups by age, sex, smoking, alcohol consumption, and “three highs” (hypertension, hyperglycemia, hyperlipidemia) history, with all OR values >1.0. Interaction tests yielded *P* for interaction values >0.05 (range 0.176–0.910), indicating robustness of the association without significant modification by these factors. Notably, in the subgroup with reduced cardiac function (Echo_EF: Low, n = 66), the predictive effect was markedly stronger, with OR = 3.79 (95% *CI*: 1.08–13.23), suggesting heightened sensitivity to “inflammation–metabolism” dual stress in patients with impaired cardiac function.

### 3.5 Predictive model performance and comparative analysis

#### 3.5.1 Model performance and internal validation

Four sequential models incorporating clinical variables were constructed. Discrimination improved progressively, with AUC increasing from 0.58 (95% *CI*: 0.54–0.63) in Model 1 to 0.79 (95% *CI*: 0.75–0.82) in Model 4. According to DeLong’s test for within-model comparisons, the improvement in AUC was statistically significant from Model 1 to Model 2 and from Model 2 to Model 3 (all *P* < 0.05). However, no further significant improvement was observed from Model 3 to Model 4 for hs-CRP-TyG (*P* > 0.05), whereas TyG showed a modest but statistically significant increase (*P* < 0.05) (**Table 3**).

**Table 3:** Discriminative performance and internal validation of hs-CRP-TyG and TyG models for coronary artery disease.

Model	Index	AUC (95%*CI*)	Bootstrap C-index (95% *CI*)	Sensitivity (%)	Specificity (%)	Optimal cutoff	*P*-value*
Model 1	hs-CRP-TyG	0.58 (0.54-0.63)	0.59 (0.53–0.64)	68	47	0.85	–
Model 2	hs-CRP-TyG	0.76 (0.73-0.80)	0.76 (0.71–0.81)	76	69	0.83	<0.01
Model 3	hs-CRP-TyG	0.78 (0.75-0.82)	0.77 (0.73–0.82)	67	79	0.88	<0.05
Model 4	hs-CRP-TyG	0.79 (0.75-0.82)	0.77 (0.72–0.82)	68	79	0.88	>0.05
Model 1	TyG	0.56 (0.52-0.61)	0.57 (0.51–0.63)	47	67	0.87	–
Model 2	TyG	0.76 (0.73-0.80)	0.76 (0.71–0.81)	77	69	0.83	<0.01
Model 3	TyG	0.78 (0.75-0.82)	0.78 (0.73–0.82)	70	79	0.87	<0.05
Model 4	TyG	0.79 (0.75-0.82)	0.77 (0.72–0.82)	68	79	0.88	<0.05

AUC indicates area under the receiver operating characteristic curve. Bootstrap C-index was calculated based on 1000 bootstrap resampling iterations to evaluate internal model discrimination. *P-values represent DeLong test comparisons between the current model and the preceding model within the same index (e.g., Model 2 vs Model 1). hs-CRP-TyG, hs-CRP–Triglyceride-Glucose; TyG, triglyceride glucose index; CI, confidence interval. Model 1, Unadjusted (crude) model; Model 2, Adjusted for age, sex, smoking history, and alcohol consumption; Model 3, Further adjusted for hypertension, diabetes mellitus, and pre-admission lipid-lowering medication use; Model 4, Fully adjusted model additionally including uric acid, fasting blood glucose, lymphocyte ratio, fibrinogen, and HDL-C.

**Figure 3:**
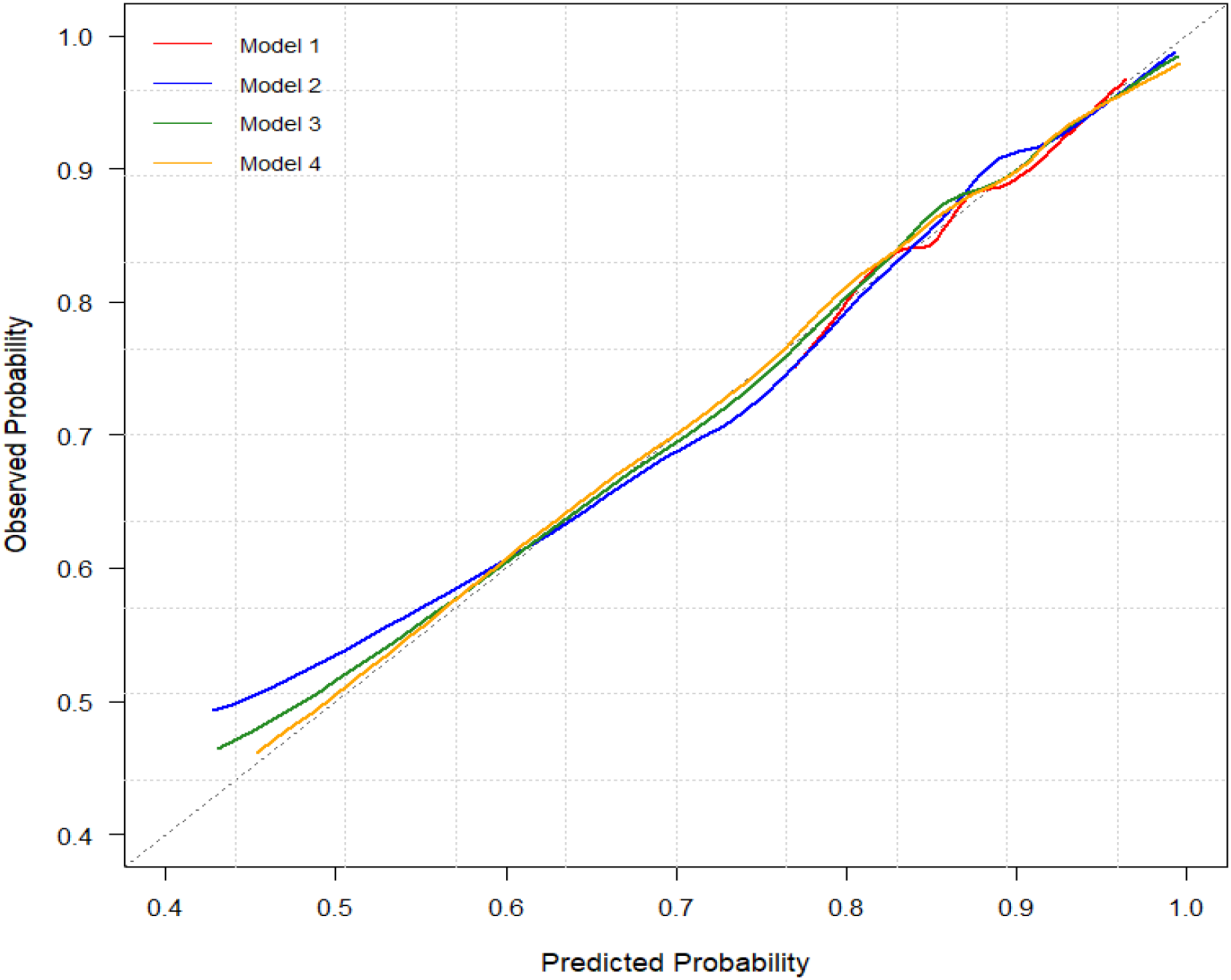
Calibration plots for predicted vs. observed probability of CAD across four models. This plot assesses the calibration accuracy of the four logistic regression models (Model 1 to Model 4) by comparing predicted probabilities (x-axis) against observed outcomes (y-axis). The diagonal dotted line represents perfect calibration (predicted = observed). Curves closer to the diagonal indicate better calibration. CAD, coronary artery disease; Models 1–4 represent progressively adjusted regression models, ranging from the unadjusted model (Model 1) to the fully adjusted model including all covariates (Model 4).

**Figure 4:**
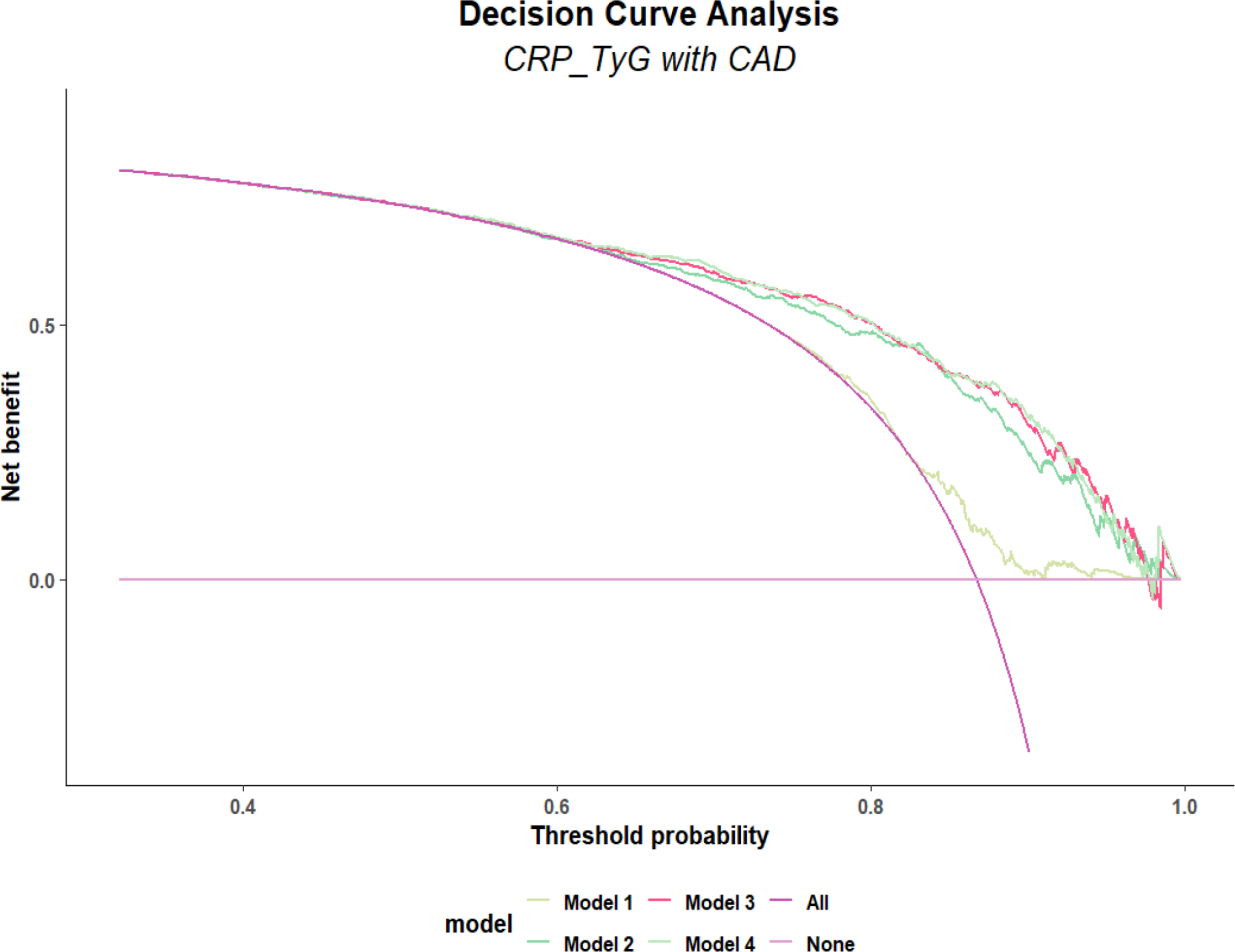
Decision curve analysis evaluating the clinical utility of the hs-CRP-TyG index for predicting CAD. The x-axis represents the threshold probability, defined as the minimum probability of disease at which a clinician would recommend further intervention. The y-axis represents the net benefit. The DCA curves for the hs-CRP-TyG models (Models 1–4) are plotted alongside the "Treat All" and "Treat None" strategies. The results show that using the hs-CRP-TyG index to guide decision-making yields a higher net benefit than the "Treat All" or "Treat None" strategies across a moderate range of threshold probabilities. This suggests that the index has potential clinical utility for risk stratification, aiding in the identification of high-risk patients who may benefit from further evaluation. hs-CRP-TyG, hs-CRP–Triglyceride-Glucose; CAD, coronary artery disease. Models 1–4 represent progressively adjusted regression models, ranging from the unadjusted model (Model 1) to the fully adjusted model including all covariates (Model 4).

Calibration performance remained stable across all models, with Hosmer–Lemeshow test *P* values > 0.05 (**Figure 3**). Bootstrap resampling (1000 iterations) demonstrated stable discrimination performance, with C-index values ranging from 0.76 to 0.77 across Models 2–4, indicating good internal validity (**Table 3**). Decision curve analysis suggested a potential net clinical benefit across a range of threshold probabilities (0.4–0.9), with no obvious separation between Model 3 and Model 4, suggesting similar clinical utility between these two models (**Figure 4**). This relatively high threshold range likely reflects the high-risk nature of the coronary angiography population, in which the pre-test probability of CAD is elevated.

#### 3.5.2 Reclassification performance

Reclassification analysis showed that hs-CRP-TyG significantly improved risk classification compared with TyG in Model 1 (NRI = 0.245, *P* < 0.01; IDI = 0.005, *P* < 0.01). However, no significant improvement was observed in Models 2 and 3 (all *P* > 0.05), and a negative NRI and IDI were observed in Model 4 (NRI = −0.223, *P* < 0.01; IDI = −0.002, *P* < 0.05), suggesting limited incremental predictive value with increasing model complexity (**Table 4**).

**Table 4:** Comparative performance and reclassification between hs-CRP-TyG and TyG models.

Model	AUC (hs-CRP-TyG)	AUC (TyG)	*P*-value*	NRI (95% *CI*)	*P*-value	IDI (95% *CI*)	*P*-value
Model 1	0.58	0.56	0.01	0.245 (0.089– 0.399)	<0.01	0.005 (0.002– 0.007)	<0.01
Model 2	0.76	0.76	0.71	-0.104 (-0.260– 0.052)	>0.05	-0.003 (-0.003– 0.002)	>0.05
Model 3	0.78	0.78	0.57	-0.151(-0.306– 0.005)	>0.05	0.001 (-0.002– 0.002)	>0.05
Model 4	0.79	0.79	0.19	-0.223(-0.376– -0.069)	<0.01	-0.002 (-0.0033– -0.001)	<0.05

AUC, area under the receiver operating characteristic curve; NRI, net reclassification improvement; IDI, integrated discrimination improvement; CAD, coronary artery disease; hs-CRP-TyG, high-sensitivity C-reactive protein–triglyceride-glucose index; TyG, triglyceride-glucose index; CI, confidence interval. *P-values were derived from DeLong’s test for pairwise comparison of AUCs between hs-CRP-TyG–based and TyG-based models. NRI and IDI were calculated to assess the incremental reclassification performance of hs-CRP-TyG relative to TyG using predefined risk categories of 0.2 and 0.6.

#### 3.5.3 Comparison with TyG index

**Figure 5:**
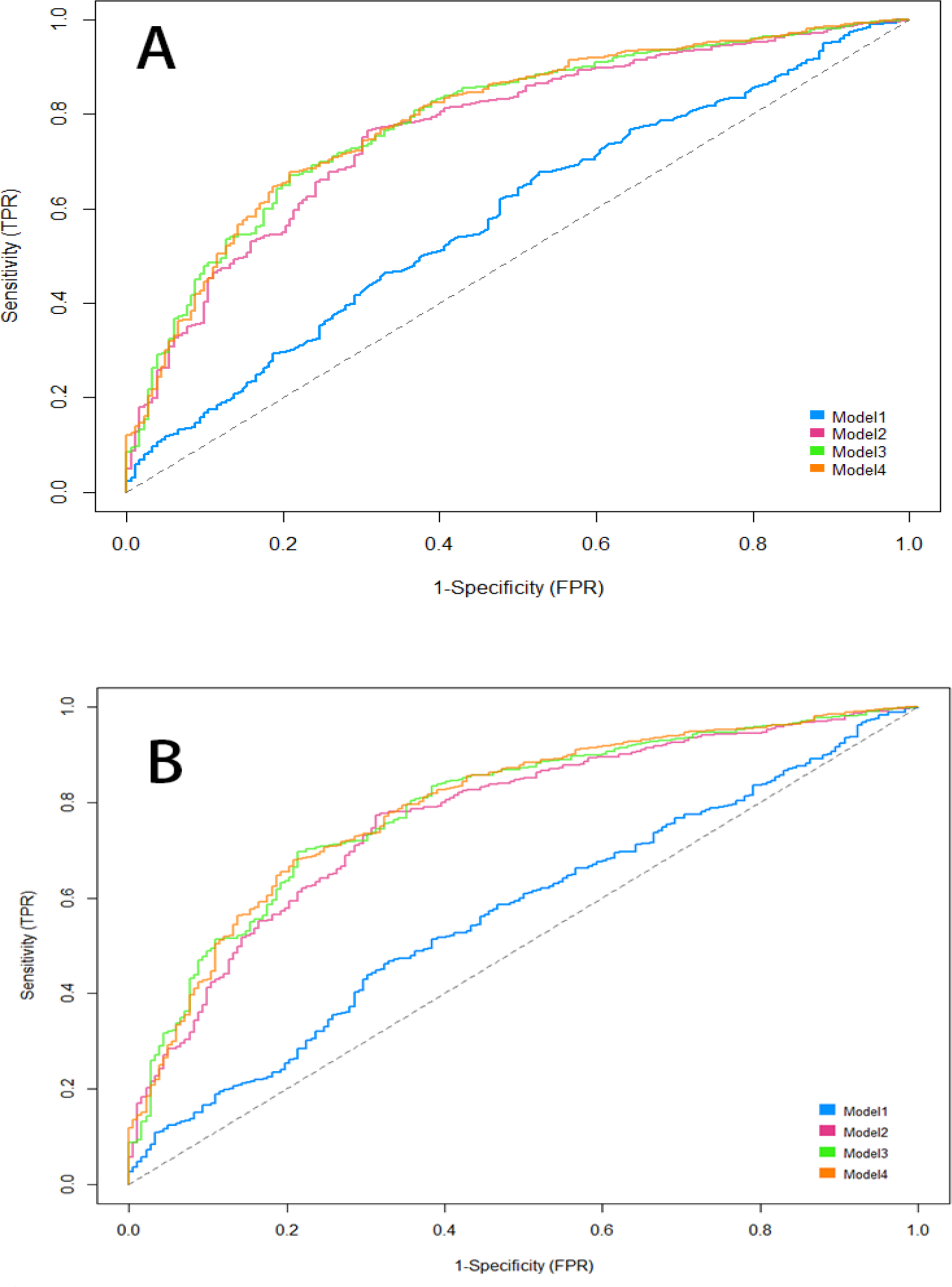
ROC curves for predicting CAD using hs-CRP-TyG and TyG indices. **(A)** ROC curve of the hs-CRP-TyG in predicting CAD risk across four multivariable models (Model 1 to Model 4). **(B)** ROC curve of the TyG index alone in predicting CAD risk across four multivariable models (Model 1 to Model 4). The diagonal dashed line represents the reference line (AUC = 0.5, no discrimination). Model 1, unadjusted; Model 2, adjusted for age, sex, smoking, and alcohol; Model 3, further adjusted for hypertension, diabetes, and lipid-lowering medication; Model 4, fully adjusted, including uric acid, lymphocyte ratio, fibrinogen, and HDL-C. hs-CRP-TyG, hs-CRP–Triglyceride-Glucose; TyG, triglyceride-glucose index; CAD, coronary artery disease; ROC, receiver operating characteristic; AUC, area under the curve; FPR, false positive rate (1-Specificity); TPR, true positive rate (Sensitivity).

In head-to-head comparisons, hs-CRP-TyG and TyG demonstrated comparable discrimination in Models 2–4 (all *P* > 0.05, DeLong test). A small but statistically significant difference was observed in Model 1 (0.58 vs. 0.56; *P* = 0.01), which was not sustained after adjustment (**Table 4**, **Figure 5**).

## 4 Discussion

In this relatively large coronary angiography cohort, the hs-CRP-TyG index exhibited a distinct graded association with anatomical CAD severity. Our findings suggest that hs-CRP-TyG is independently associated with CAD occurrence and is more strongly related to severe and extremely severe stenosis, whereas no significant association was observed in mild stenosis. Subgroup analyses further supported the robustness of this association across diverse populations.

Compared with the TyG index alone, hs-CRP-TyG showed higher sensitivity in the unadjusted model and comparable discriminative performance across adjusted models. However, this advantage was not maintained in fully adjusted models, indicating limited incremental value beyond established clinical and biochemical factors.

These findings suggest a potential link between metabolic dysfunction and inflammation in CAD. The association between hs-CRP-TyG and CAD may reflect the interaction between chronic low-grade inflammation and insulin resistance, where inflammatory activity impairs insulin signaling and metabolic disturbance further promotes inflammatory responses through pathways such as NF-κB (22). These mechanisms remain hypothesis-generating rather than causal.

Notably, hs-CRP-TyG was significantly associated with severe stenosis (L2/L3), suggesting that elevated metabolic–inflammatory burden may promote plaque instability through smooth muscle cell apoptosis, MMP activation, and fibrous cap thinning (23). In contrast, mild stenosis (L1) may be driven primarily by local hemodynamic or lipid-related factors and appears less sensitive to systemic inflammation (24). These interpretations remain speculative and require further experimental validation. These findings are consistent with a potential role of metabolic–inflammatory burden in advanced CAD phenotypes (25).

The observed sensitivity advantage of hs-CRP-TyG over TyG may reflect the heterogeneity of CAD pathogenesis, in which a subset of patients exhibit an “inflammation-dominant” phenotype characterized by chronic inflammatory activation while maintaining relatively preserved glucose–lipid metabolism, making them potentially less detectable by TyG alone (26). By incorporating inflammatory status, hs-CRP-TyG may improve risk identification in high-risk clinical settings. However, this advantage diminishes after adjustment for additional clinical and biochemical variables, consistent with the observed plateau in model performance, suggesting limited incremental prognostic information beyond established clinical and biochemical risk factors.

We identified a nonlinear dose–response relationship between hs-CRP-TyG and CAD risk, with an inflection point around 1.42, beyond which risk increased more rapidly, particularly for severe stenosis. This threshold may aid risk stratification, but requires prospective validation before clinical application.

Regarding confounding control, ABI and LVEF were included to account for systemic atherosclerotic burden and cardiac function, respectively. ABI is a marker of systemic vascular disease and is independently associated with CAD risk, while LVEF reflects overall cardiac functional status and may be influenced by metabolic and inflammatory processes (27, 28). However, these variables may lie on shared causal pathways, introducing potential overadjustment bias. Therefore, while model completeness is improved, effect estimates should be interpreted cautiously. This approach is consistent with methodological recommendations emphasizing inclusion of clinically relevant confounders to improve robustness of observational estimates (29).

These findings may have potential clinical implications. First, hs-CRP-TyG may serve as a simplified tool for preliminary risk stratification, particularly in outpatient or resource-limited settings. However, it should not be interpreted as a standalone indication for coronary imaging, which must be guided by clinical presentation, comorbidities, and established guideline-based assessment. Second, the association between hs-CRP-TyG and severe stenosis suggests a potential role in identifying higher-risk patients. Nevertheless, its value in guiding therapeutic decisions, including revascularization strategies or targeted therapies, remains uncertain and requires prospective validation.

External validation in independent, multicenter cohorts is required before clinical implementation, given the single-center and high-risk nature of the study population. Strengths of this study include the use of coronary angiography as a reference standard, a relatively large sample size, and comprehensive statistical analyses including internal validation and reclassification metrics. Several limitations should be acknowledged. First, the retrospective cross-sectional design precludes causal inference. Second, the high proportion of CAD cases reflects a selected population undergoing coronary angiography, which may introduce spectrum bias and potentially overestimate diagnostic performance. Third, the CAD definition was based on anatomical stenosis without functional assessment (e.g., FFR or iFR), which may limit clinical interpretation. Fourth, the study was conducted in a single center, and external validation in independent cohorts is required. Finally, although NRI and IDI analyses were performed, the incremental value of hs-CRP-TyG diminished in fully adjusted models, suggesting limited added benefit beyond conventional markers.

## 5 Conclusion

In conclusion, hs-CRP-TyG is associated with both the presence and severity of CAD and may reflect the combined effects of metabolic and inflammatory processes. While it shows potential for risk stratification in high-risk clinical settings, its additional predictive value beyond established clinical variables appears limited in fully adjusted models. Further prospective and multicenter studies are needed to confirm its clinical utility.

## Data Availability

The data analyzed in this study is subject to the following licenses/restrictions: The data for this study were sourced from the Chongqing University Fuling Hospital. This dataset is not publicly available, but it can be obtained upon reasonable request from Min Yang (yyyflyy@163.com).
